# Comparative study of ovarian development in wild and captive-reared long-whiskered *Sperata aor* (Hamilton, 1822)

**DOI:** 10.1186/s40850-023-00172-x

**Published:** 2023-07-24

**Authors:** Muhammad Anamul Kabir, Mohammed Mahbub Iqbal, Shishir Kumar Nandi, Mahbuba Khanam, Md Afsar Ahmed Sumon, Albaris B. Tahiluddin, Zulhisyam Abdul Kari, Lee Seong Wei, Guillermo Téllez-Isaías

**Affiliations:** 1 Department of Aquaculture, Sylhet Agricultural University, Sylhet, 3100 Bangladesh; 2 Department of Fish Biology and Genetics, Sylhet Agricultural University, Sylhet, 3100 Bangladesh; 3 Marine Biology Department, Faculty of Marine Sciences, King Abdulaziz University, Jeddah, Saudi Arabia; 4 College of Fisheries, Mindanao State University-Tawi-Tawi College of Technology and Oceanography, Sanga-Sanga, Bongao, 7500 Tawi-Tawi Philippines; 5 Department of Agricultural Sciences, Faculty of Agro-Based Industry, Universiti Malaysia Kelantan, Jeli Campus, Jeli, 17600 Kelantan Malaysia; 6 Advanced Livestock and Aquaculture Research Group, Faculty of Agro-Based Industry, Universiti Malaysia Kelantan, Jeli Campus, Jeli, 17600 Kelantan Malaysia; 7 Department of Poultry Science, University of Arkansas, Fayetteville, AR 72701 USA

**Keywords:** Broodstock, Oocytes, Ovarian histology, Amino acid, Sustainable aquaculture

## Abstract

Long-whiskered catfish *Sperata aor* is a freshwater catfish known for its supreme flesh quality and fast growth, whose captive-reared broodstock denotes a difficult challenge for aquaculture. The reproductive dysfunctions in long-whiskered catfish raised in tank conditions were observed by comparing tissue biochemical composition and ovarian histology of wild female broodstock. Sixty (60) female broodstocks were used in the current study, consisting of 30 reared at sandy-muddy soil tank bottoms in captive conditions and 30 wild individuals collected from the haor basin during the breeding season. The fish reproductive state was investigated using the biometric and reproductive parameters, biochemical composition and levels of amino acids in the different tissues, and histological analysis of ovarian development. Results revealed that the biometrical parameters of wild and captive female broodstocks exhibited no remarkable difference (p > 0.05). Nevertheless, the wild fish had remarkably higher (p < 0.05) GSI (8.73%), oocyte weight (0.45 mg/egg), and ripeness (27.08%) in comparison with captive-reared broodstock. The total length and body weight, body weight and ovary weight, ovipositor diameter and ovary weight, and GSI and HSI displayed a positive relationship with R^2^ = 1, R^2^ = 1, R^2^ = 0.993, and R^2^ = 0.973, respectively, for wild broodstock, while R^2^ = 0.994, R^2^ = 0.806, R^2^ = 0.804, and R^2^ = 0.896, respectively, for captive broodstock. Additionally, the proximate composition in oocytes and liver tissues in both broodstocks did not differ significantly (p > 0.05). However, two essential amino acids (EAA), i.e., lysine and phenylalanine, and two non-essential amino acids, i.e., glutamic acid and glycine, were highly significant differences (p < 0.05) in the oocytes and liver of wild broodstock compared to the captive-reared broodstock. On the other hand, the EAA, e.g., isoleucine, threonine, leucine, and arginine, were highly dominated in both wild and captive female brood oocytes and liver. The ovarian histological slides from each fish group showed three oocytes developmental stages that indicated the asynchronous-reproductive ovarian oocytes of this fish. This study may be useful to fully understand the factors affecting the spawning and reproduction of *S. aor* broodstock, crucial for management in captive conditions as well as conservation and protection for sustainable aquaculture management of *S. aor*.

## Introduction

Long-whiskered *S. aor* (Hamilton, 1822) is a large freshwater epibenthic teleost catfish with a wide range of geographical distribution which include Bangladesh, India, Pakistan, Nepal, and Myanmar [1, 2]. This fish is a gonochoric species with asynchronous ovarian oocyte development and has several spawning times during the reproductive season [3, 4]. Long-whiskered catfish has been deemed among the most favored edible fish due to its rapid growth, excellent flesh quality, low number of intramuscular bones [1, 5], and great nutritional value with quality protein content [1, 6]. Recently, *S. aor* has a tremendous market demand in Bangladesh, triggering a growing interest in aquaculture production of this fish species.

However, this fish is now considered an endangered species owing to the substantial depletion of the natural breeding and feeding grounds along with climate change as well as human intervention [7–9]. Its domestication represents an effective tool [10] to protect from extinction as well as increase aquaculture production and competitiveness, hence providing protein security to human and sustainable aquaculture management of *S. aor* in the haor basin of Sylhet. However, it is an ambitious challenge for carnivorous fish to rear in captivity, especially when confined in tanks or earthen ponds, as they may manifest significant reproductive dysfunctions [10–12]. Additionally, the *S. aor* broodstock has not yet spawned in captive conditions but reproduced in nature, specifically in the freshwater haor basin and river. Therefore, the aquaculture sector requires innovative and sustainable research for the development of hormone-induced breeding strategies to meet the larval requirement of that fish for large-scale industrial development. On the other hand, many previous studies reported that the reproductive performance and oocytes quality in broodstock are influenced by numerous factors, such as housing and tank bottom condition [4], stress, genetics, nutritional status, egg over-ripening, broodstock age, environmental and water quality factors [13–16].

Previous studies [17, 18] stated that protein and amino acid are crucial bimolecular components, regulating key metabolic pathways and serving as precursors for the synthesis of biologically active substances for oocytes or eggs formation in the ovary of broodstock. Moreover, protein and lipid are the main components of egg yolk, which are actively utilized by broodstock as nutrient sources for reproductive development and early embryogenesis [11, 19]. Amino acid groups, especially isoleucine, leucine, glutamic acid, valine, alanine, lysine, serine, and aspartic acid, have a significant role in broodstock reproductive development, as reported by previous researchers [11, 15, 20, 21]. On the other hand, light microscopic observation of ovarian histology indicates the reproductive status and oocyte development stages of broodstock in the reproductive period [22, 23].

Many studies exist on the comparative analysis of wild and captive broodstock reproductive performance [10, 24, 25], biochemical composition [10, 26–30], amino acid profile [26, 28], and ovarian histological investigation [4, 10] in different species of fish. However, there is a lack of comparative analysis of reproductive development and tissue biochemical composition in the wild and captive *S. aor* female broodstock. Hence, the current work aimed to study the variability in reproductive development, ovary and liver biochemical and amino acid composition, and oocytes histological analysis in wild and captive female *S. aor* broodstock.

## Materials and methods

### Broodstock collection and husbandry condition

Sixty (60) female individuals (30 wild and 30 captive-reared) of long-whiskered catfish were used for this experiment. Six months before, sexually matured broodstocks were collected from the haor basin in late April to early May in 2020 and immediately brought to the Aquaculture Laboratory and then stocked in a large fiberglass tank (1000 L). A commercial diet (34% crude protein, 6% crude lipid, 18% ash, and 8% fibre-Saudi-Bangla Fish Feed Limited, Bangladesh) was used for feeding the fish until apparent satiation, given twice daily at 09.30 and 17.00 over 90 days of the experiment. On the other hand, wild broodstocks were collected from a fish landing center of the haor basin during the peak breeding season in late July to early August in 2020.

### Biometrical and reproductive variables

For the analysis of the biometrical and reproductive variables, 10 broodstocks from each group were immediately transferred to the laboratory and subjected to anesthesia with MS_222_ at a dose of 0.1 g/L water. In each broodstock, ovipositor color was observed, and ovipositor diameter, body weight, and total length were recorded. After that, each broodstock’s body cavity was opened, and the liver, viscera, ovary, and fat were taken out and separately weighed. Oocytes, ovary weight, oocyte weight, relative fecundity, and oocyte ripeness were measured according to Kabir et al. [11] and Nandi et al. [19]. Moreover, the fish biometric and reproductive parameters were evaluated by using the following formulae:

i. Hepatosomatic index (HSI) = (Liver weight/ Body weight) x 100.
ii. Visceral somatic index (VSI) = (Viscera weight/ Body weight) x 100.
iii. Gonadosomatic index (GSI) = (Gonad weight/ Body weight) x 100.
iv. Intra-peritoneal fat (IPF) = (Fat weight/ Body weight) x 100.
v. Condition factor (CF) = Final weigh (g)/ (Fish total length, cm)^3^.
vi. Fecundity (eggs/ female BW) = (Total number of oocytes in female ovary/ Body weight).
vii. Ripe oocytes (%) = Number of oocytes with yolk position near one edge of the oocyte/ Total number of oocytes counted) x 100.

### Biochemical composition of liver tissues and oocytes

The proximate composition of broodstock liver tissues and oocytes was performed by employing the standard protocol of AOAC [31] with few modifications. Shortly, the Kjeldahl method was utilized to analyze crude protein (% N x 6.25), the Soxhlet apparatus was used to measure crude lipid by n-hexane extraction, and the Muffle furnace was used to determine ash after incinerating the sample at 550 °C for 6 h. Triplicate determinations were carried out on each sample analysis.

### Amino acid profile in the liver and oocytes tissues

Analysis of fish liver and ovary tissues’ amino acids was carried out according to a standard procedure reported by Kabir et al. [11] and Kari et al. [32] with some modifications. Briefly, the test samples from each broodstock group were gathered and then refrigerated at -20°C until subsequent analysis. Each test sample’s amino acid content was assessed after hydrolyzing with 6 N HCL by heating at 110°C for 24 h, and then derivatization with AccQ Fluor reagent (6-aminoquinolyl-N-hydroxysuccinimdylcarbamate) was performed, followed by chromatographic separation employing an AccQTagTM reversed phase (RP) analytical column (3.9 × 150 mm, length x inner diameter). The HPLC (High-Performance Liquid Chromatography) system with a Waters 1525 Binary HPLC Pump, 717 Plus auto-sampler (R), and Waters 2475 Multi λ Fluorescence detector (250 nm wavelength, 395 nm emission), was employed to determine the amino acid profile. For quantitative determination, the α-amino butyric acid (AABA) was utilized as an internal standard. In addition, Acetonitrile and AccQTagTM were utilized as eluents. Integration, identification, and quantification of chromatographic peaks were made with the use of BreezeTM software version 3.20 by comparing them with known standards (Amino acid standard H, Pierce, Rockford, Illinois, USA). In the present study, amino acids, such as cysteine, methionine, and sulfur amino acids, were not investigated, and verified the test samples with three replicates.

### Histological investigation of ovary

The histomorphological investigation of broodstock ovary was performed according to minor modifications of the Saxena et al. [33] method. Shortly, a total of 4 fish from each group were selected randomly and anesthetized with MS_222_ at a dose of 0.1 g/L water. The fish was then cut ventrally, and transverse sections of the ovary’s anterior, middle, and posterior parts were collected and preserved in 10% neutral buffered formalin. Then, each transverse section was processed and dehydrated in graded ethanol series, embedded in a paraffin block, sectioned (8 μm), and stained in hematoxylin and eosin (H & E) solution. The histological slides were observed under a compound light microscope (Olympus BX43), and a digitalized camera (Olympus Xcam-Alpha, Germany) was used to capture microphotographs. Furthermore, the classification of various phases of oocyte development in the broodstock ovary was performed according to the method used previously [4, 34]. In brief, this study involved the examination of ten ovarian oocytes histological slides from wild and captive broodfish under a light microscope (Olympus BX43). Then, the total number of oocytes within each stage of development was measured based on the morphological features like presence of nucleus, yolk granule or vesicle and the average values were determined. Subsequently, the frequency percentage distribution was calculated by dividing the oocyte counts in each stage by the total number of oocytes counted and multiplying the values by 100.

### Environmental parameters

The monthly mean temperature and rainfall data were collected from the meteorological department of Sylhet divisional office from January to December in 2020.

### Statistical analysis

The female broodstock’s biometrical, reproductive, proximate, and amino acids composition, oocyte development stages, and environmental data were analyzed by the SPSS software version 26.0 using an independent sample t-test with the mean values for comparing the wild and captive broodstock. Significant differences were considered when p < 0.05. Presented data are in the form of mean ± standard deviation (SD).

## Results

### Biometrical and reproductive parameters

Table 1 displays the biometrical and reproductive variables of wild and captive female *S. aor* broodstock. The mean values of total length, body weight, HSI, VSI, IPF, and CF of wild and captive broodstock were not remarkably varied (p > 0.05) in this study. Similar to this, the reproductive indices in terms of ovary weight, ovipositor diameter, and fecundity did not exhibit any significant variances (p > 0.05) between broodstock groups. However, the significantly highest (p < 0.05) GSI, oocyte weight, and egg ripeness were found in wild broodstock as compared to broodstock raised in captivity with numerical values of 8.73 ± 0.06, 0.45 ± 0.03 and 27.08 ± 3.33, respectively. Moreover, the ovipositor color of both wild and captive broodstock ranged from reddish to pinkish.

**Table 1:** Biometrical and reproductive indices of female *S. aor* broodstock (n = 30). Data presented are expressed as mean ± SD.

Parameters	Wild	Captive	p-value
Total length (cm)	37.81 ± 1.67	37.71 ± 1.67	0.82
Body weight (g)	377.46 ± 16.67	374.43 ± 16.71	0.49
Ovary weight (g)	32.81 ± 1.67	32.71 ± 1.67	0.82
OD (cm)	0.55 ± 0.08	0.54 ± 0.08	0.65
GSI	8.73 ± 0.06^Sig^	8.69 ± 0.06^Sig^	0.01
HSI	1.48 ± 0.16	1.46 ± 0.16	0.70
VSI	3.4 ± 0.38	3.57 ± 0.62	0.24
IPF	1.16 ± 0.08	1.18 ± 0.14	0.43
CF (g/cm^3^)	0.70 ± 0.06	0.70 ± 0.06	0.98
Fecundity (eggs/ female) X 10^3^	73.84 ± 28.90	73.81 ± 28.89	1.00
Oocyte weight (mg)	0.45 ± 0.03^Sig^	0.37 ± 0.04^Sig^	0.00
Oocyte ripeness (%)	27.08 ± 3.33^Sig^	12.65 ± 3.05^Sig^	0.00
Ovipositor color	Reddish to pinkish	Reddish to pinkish	

OD: Ovipositor diameter, GSI: Gonadosomatic index, HSI: Hepatosomatic index, VSI: Visceral-somatic index, IPF: Intraperitoneal fat, CF: Condition factor. *Sig indicates significant differences (p < 0.05) between the broodstock groups.

### Relationship between biometric and reproductive variables

**Fig. 1:**
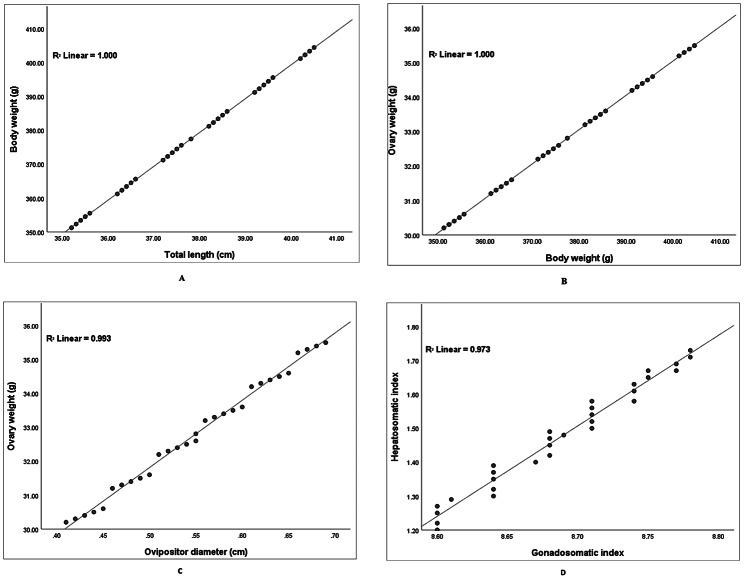
**A**. Relationship between total length and body weight, **B**. Body weight and ovarian weight, **C**. Ovipositor diameter and ovarian weight, and **D**. GSI and HSI of wild female *S. aor*

**Fig. 2:**
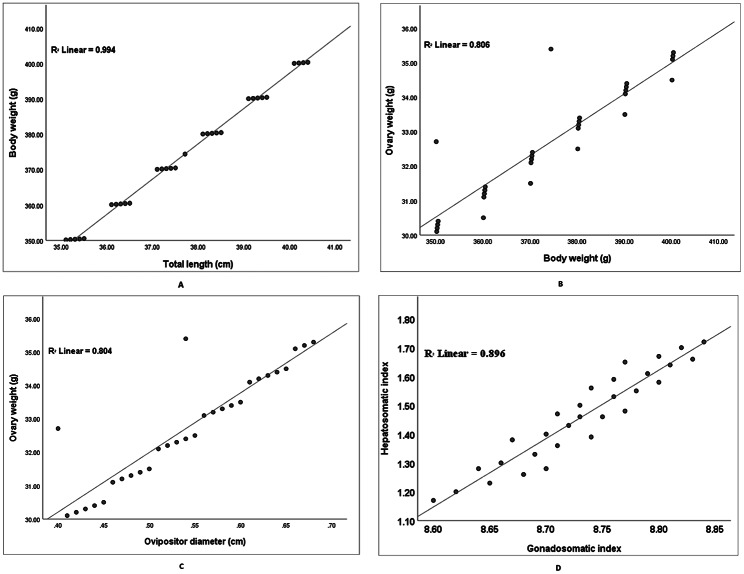
**A**. Relationship between total length and body weight, **B**. Body weight and ovarian weight, **C**. Ovipositor diameter and ovarian weight, and **D**. GSI and HSI of captive female *S. aor*

The linear regression relationship between biometrical and reproductive parameters of wild and captive broodstocks is illustrated in Figs. 1 and 2. The body weight and total length of wild and captive-reared fish displayed a strong positive relationship (R^2^ = 1.000 and 0.994, respectively). Similarly, both body weight and ovipositor diameter were highly correlated with their o`varian weight, with R^2^ = 1.000 and 0.993, respectively, for wild fish and R^2^ = 0.806 and 0.804, respectively, for captive fish. Additionally, the broodstock GSI had a strong correlation with HSI, with R^2^ values of 0.973 and 0.896, respectively, for wild and captive fish.

### Biochemical composition of liver and oocytes tissues

The proximate composition of the liver and oocytes of captive and wild broodstock are shown in Table 2. In this investigation, there had no substantial differences (p > 0.05) between the captive and wild fish tissues’ proximate composition, such as protein, lipid, and ash content. However, liver and oocytes from wild broodstock had numerically higher protein and lipid than those from captive female broodstock. In addition, the oocytes contained a higher amount of ash in each broodstock group compared with the liver tissue of both condition broodstock.

**Table 2:** Proximate composition (% dry matter basis) of female *S. aor* broodstock liver and oocytes (n = 3). Data presented are expressed as mean ± SD.

Parameters	Wild	Captive	p-value
Liver			
Protein	51.31 ± 1.73	50.33 ± 1.61	0.51
Lipid	25.54 ± 0.50	25.66 ± 0.70	0.82
Ash	4.66 ± 0.33	4.54 ± 0.31	0.65
Oocytes			
Protein	61.63 ± 0.55	61.33 ± 0.66	0.57
Lipid	29.03 ± 0.84	28.74 ± 1.09	0.74
Ash	5.77 ± 0.21	5.99 ± 0.14	0.22

### Amino acid composition of liver

The amino acid profile of the liver from the wild and captive-reared broodstock is shown in Table 3. The amino acid deposition in the liver exhibited no remarkable differences (p > 0.05) between the test fish groups, except for some cases. The amino acids such as lysine, phenylalanine, and glycine were remarkably varied (p < 0.05) between the broodstock groups, and their highest (p < 0.05) values were noted in the wild fish liver. Threonine, isoleucine, leucine, and lysine were the most predominant essential amino acids (EAAs), while glutamic acid and glutamine predominated among nonessential amino acids (NEAAs) in both fish groups.

**Table 3:** Amino acid composition (% dry matter basis) of liver tissue of female *S. aor* broodstock (n = 3). Data presented are expressed as mean ± SD.

Amino acids	Wild	Captive	p-value
**EAA**			
Threonine	4.50 ± 0.50	4.50 ± 0.50	1.00
Valine	2.42 ± 0.01	2.43 ± 0.01	0.28
Asparagine	2.79 ± 0.01	2.78 ± 0.01	2.56
Isoleucine	4.77 ± 0.01	4.48 ± 0.01	0.56
Leucine	5.50 ± 0.10	5.50 ± 0.15	0.56
Histidine	2.42 ± 0.01	2.43 ± 0.01	0.28
Lysine	10.49 ± 0.01^Sig^	5.56 ± 0.15^Sig^	0.00
Arginine	3.46 ± 0.00	3.45 ± 0.01	0.06
Tryptophan	2.79 ± 0.01	2.78 ± 0.01	0.56
Phenylalanine	3.66 ± 0.01^Sig^	3.34 ± 0.01^Sig^	0.00
**NEAA**			
Aspartic acid	2.79 ± 0.01	2.78 ± 0.01	0.56
Serine	5.72 ± 0.01	5.72 ± 0.01	0.56
Glycine	4.98 ± 0.01^Sig^	4.89 ± 0.00^Sig^	0.02
Glutamic acid	12.00 ± 1.00	13.00 ± 1.00	0.28
Alanine	2.42 ± 0.01	2.43 ± 0.01	0.28
Tyrosine	5.72 ± 0.01	5.72 ± 0.01	0.56
Glutamine	12.00 ± 1.00	12.00 ± 1.00	0.28
Proline	2.43 ± 0.01	2.42 ± 0.00	0.28

*Significant indicates remarkable differences (p < 0.05) between broodstock groups.

### Amino acid composition of oocytes

The amino acid content in the broodstock oocytes is mentioned in Table 4. The lysine, phenylalanine, and glutamic acid were remarkably greater (p < 0.05) in the oocytes of wild broodstock with mean and standard deviations of 3.46 ± 0.01%, 3.66 ± 0.01%, and 10.49 ± 0.01%, respectively, while captive broodstock oocytes contained a significantly greater (p < 0.05) concentration of glycine (4.92 ± 0.00%). However, other amino acid profiles in the oocytes of wild and captive *S. aor* broodstock followed no discernible variations (p > 0.05). The most distributed EAAs in wild and captive broodstock oocytes were arginine, followed by isoleucine, leucine, and threonine.

**Table 4:** Amino acid composition (% dry matter basis) of oocytes of female *S. aor* broodstock (n = 3)

Amino acids	Wild	Captive	Significant differences
**EAA**			
Threonine	4.50 ± 0.50	4.50 ± 0.50	1.00
Valine	2.90 ± 0.01	2.80 ± 0.01	0.56
Asparagine	2.43 ± 0.01	2.42 ± 0.01	0.28
Isoleucine	4.72 ± 0.01	4.71 ± 0.01	0.56
Leucine	4.70 ± 0.50	4.50 ± 0.50	0.39
Histidine	2.43 ± 0.01	2.42 ± 0.01	0.28
Lysine	3.46 ± 0.01^**Sig**^	3.44 ± 0.00^**Sig**^	0.02
Arginine	5.60 ± 0.15	5.50 ± 0.10	0.56
Tryptophan	2.79 ± 0.01	2.78 ± 0.01	0.56
Phenylalanine	3.66 ± 0.01^**Sig**^	3.44 ± 0.01^**Sig**^	0.00
**NEAA**			
Aspartic acid	2.78 ± 0.01	2.79 ± 0.01	0.56
Serine	2.43 ± 0.01	2.42 ± 0.01	0.28
Glycine	4.89 ± 0.01^**Sig**^	4.92 ± 0.00^**Sig**^	0.00
Glutamic acid	10.49 ± 0.01^**Sig**^	5.50 ± 0.15^**Sig**^	0.00
Alanine	5.72 ± 0.01	5.72 ± 0.01	0.56
Tyrosine	5.72 ± 0.01	5.72 ± 0.01	0.56
Glutamine	13.00 ± 1.00	12.00 ± 1.00	0.28
Proline	2.42 ± 0.01	2.42 ± 0.01	0.28

*Sig indicates remarkable differences (p < 0.05) between broodstock groups.

### Histological analysis of ovary

**Fig. 3:**
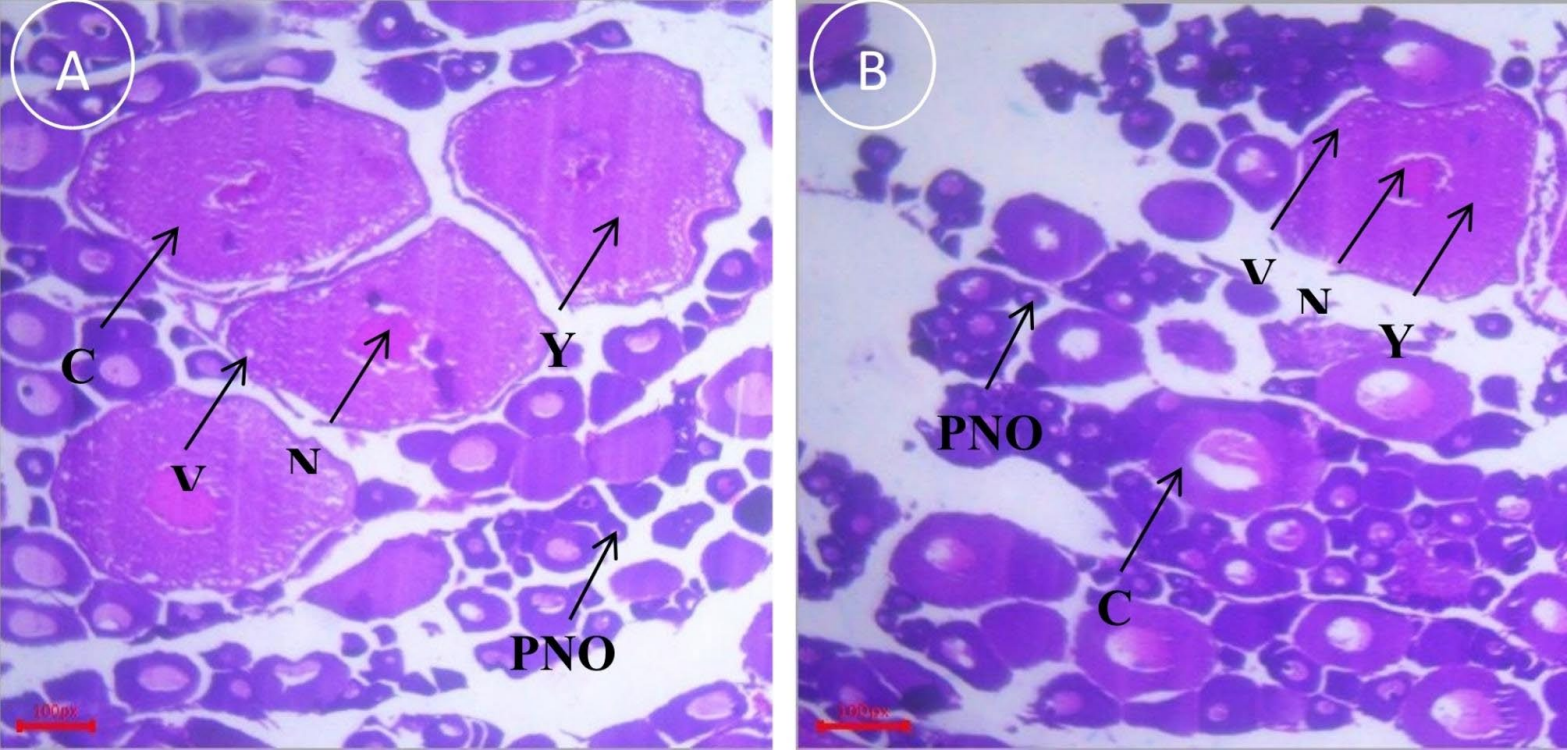
Microphotographs of ovarian histology, illustrating the oocyte development of (**A**) wild and (**B**) captive-reared *S. aor* broodstock. All photographs were captured at 10X magnification and 100 px scale bar. PNO: Peri nucleolus oocytes, YG: Yolk granulose, N: Nucleus, CY: Cytoplasm, and V: Vacuoles

The histomorphological examination of the ovary in the reproductive season demonstrated that ovary contained oocytes at various developmental stages which were divided into three groups: the peri nucleolus oocyte (PNO), yolk vesicle oocyte (YVO), and yolk granule oocyte (YGO) (Fig. 3). Additionally, the frequency percentage distribution of different stages of oocyte development in captive and wild broodstocks are summarized in Table 5. In this study, the oocyte development phases, such as PNO and YVO, were remarkably varied (p < 0.05), while YGO displayed no notable variation (p > 0.05) between the fish groups.

**Table 5:** Frequency distribution of various phases of wild and captive broodstock oocytes (n = 10)

Parameters	Wild	Captive	p-value
Primary oocyte	60.60 ± 2.22^Sig^	66.00 ± 1.70^Sig^	0.00
Yolk vesicle oocyte	22.20 ± 1.48^Sig^	18.40 ± 0.52^Sig^	0.00
Yolk granule oocyte	16.20 ± 1.62	14.60 ± 1.35	0.27

*Sig indicates remarkable differences (p < 0.05) between broodstock groups.

### Environmental parameters

**Fig. 4:**
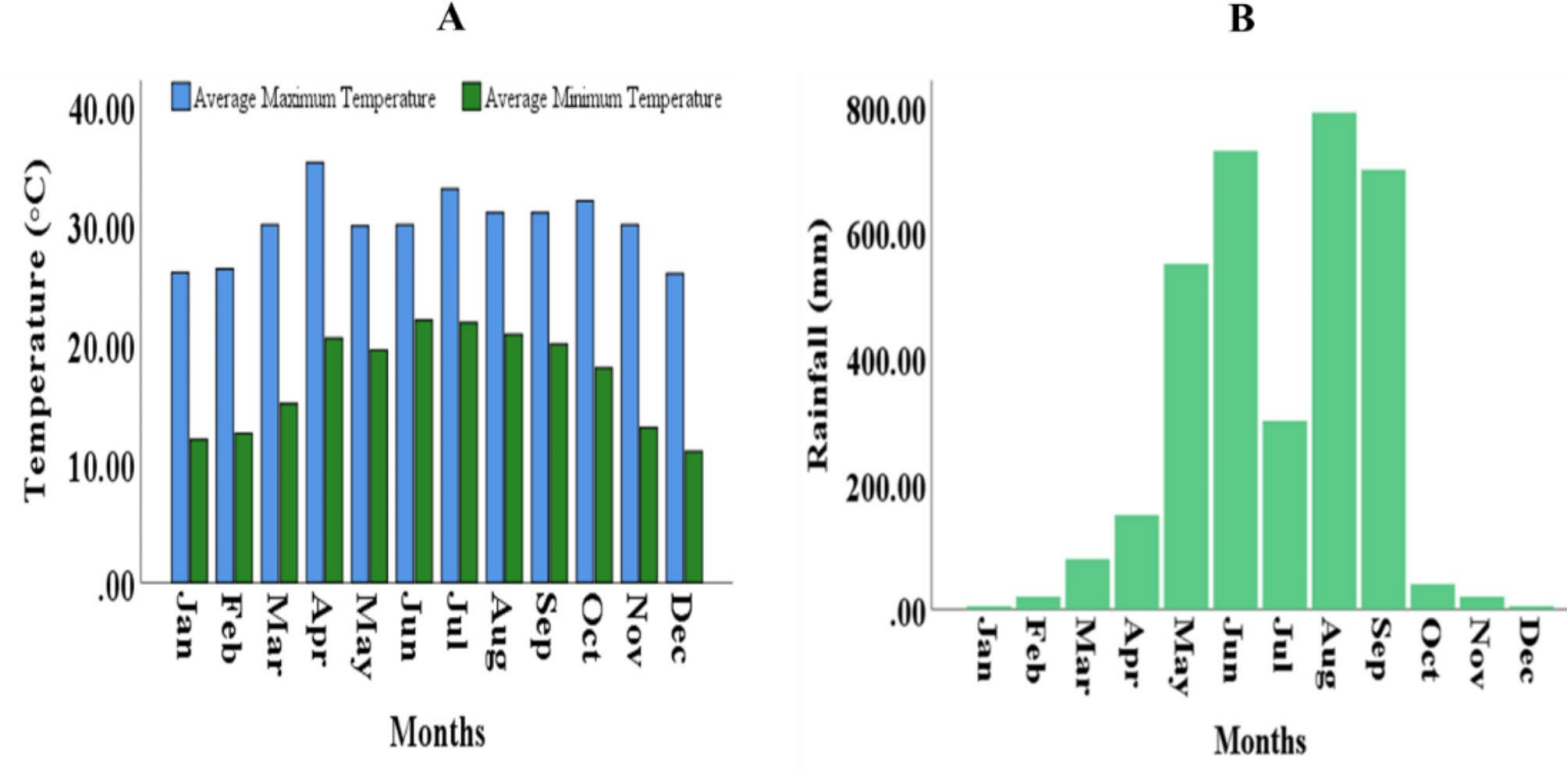
Variations of annual temperature (**A**) and rainfall (**B**) in the experimental site of Sylhet Agricultural University

Figure 4 illustrates the environmental parameters, such as average temperature and rainfall, in the experimental site of Sylhet Agricultural University between January and December, 2020. The average maximum temperature gradually rose, with April recording the highest readings (35°C). In contrast, December had the lowest average maximum temperature ever recorded (25.9°C). The total amount of rainfall rose consistently from January to April and then dramatically increased up to June before declining in the next month. The total rainfall began to rise again just after July and reached its peak in August (790.8 mm) and then drastically decreased in the following months.

## Discussion

Fish rearing conditions and environmental variations have noteworthy impacts on the reproductive state of fish, especially in long-whiskered catfish. Therefore, these findings provided insights into observing the reproductive development, egg quality, tissue biochemical composition, and ovarian histology of captive-reared and wild female *S. aor* during the reproductive season. According to the findings of this work, there were no considerable variations between the wild and farmed broodstock biometrical indices in terms of total length, body weight, HSI, VSI, IPF, and CF. The present outcomes are parallel with the previous studies’ results [35, 36]. However, the reproductive parameters, especially GSI, oocyte weight, and ripeness, were highly significant between the broodstock groups. As the broodstock used in this study were sexually matured enough (weight: 360–390 g and size: 36–38 cm) and therefore, the present results indicated that the available nutrients and energy source were utilized by fish for their reproductive and oocyte development instead of somatic growth. GSI is a valuable tool that demonstrates the reproductive progress of broodstock. A higher mean value of GSI in wild-caught broodstock suggested that those fish had better reproductive development than the fish in captivity. Zupa et al. [10] agreed with this result and found that wild *Seriola dumerili* had a significantly higher GSI during the spawning period than the captive broodstock. Similar outcomes were also noted in *Pimelodus maculatus* [37], *Melanotaenia boesemani* [38], and *Planiliza parsia* [39]. This variation of GSI may be attributed due to the discrepancy in egg weight and ripeness in the female ovary. Jabed et al. [3] reported that the uptake of liver protein, such as vitellin, into the gonad during the reproductive period significantly increases ovary weight. In parallel with our finding, previous literature [4, 36] documented that the broodstock fecundity was not greatly affected by various environmental conditions. Nevertheless, an opposite outcome was also noted in *Clarias batrachus* [24] and *Setipinna phasa* [25]. The wild and captive fish oocytes’ weight and ripeness were demonstrated to be significantly different. The equivalent outcomes were also noted in Buitta catfish *Sperata* sp [4]. Even though the fish in this trial was of identical size, the variations in some reproductive parameters might have been produced due to the environmental effects which encourage oocyte development in broodstock.

The regression result in this observation revealed that there had a strong correlation between the body weight and total length of wild and captive fish, indicating that fish length significantly increased with the body weight increment. This result is paralleled with previous reports [3, 22, 40]. The body weight and ovipositor diameter exhibited a positive relationship with ovary weight in each broodstock group. This relationship is a valuable indication of the reproductive development of broodstock with reference to its size. The oocyte output is increased with the increase in size, while larger fish have increased ovarian mass, egg diameter, and quantity [3, 41, 42]. Furthermore, Hismayasari et al. [38] revealed that the rainbow fish’s GSI and HSI values were highly correlated (R^2^ = 0.83), which is almost similar to our finding, where R^2^ > 0.89 for both test fish.

The biochemical composition of the liver and oocytes showed no significant alteration between the fish from captive and wild conditions in this study. Nonetheless, the protein and lipid content of both tissues were numerically higher in the wild than in the captive broodstock. Zupa et al. [10] published equivalent outcomes in captive and wild *Seriola dumerili* oocytes during early and advanced gametogenesis stages. The liver and oocyte nutritional profile is an egg quality indicator during the reproductive season, as the oocytes must satisfy the nutritional requirements of larval and embryonic growth [43, 44]. The liver and oocytes’ protein and lipid content also had a profound effect on broodstock size, ovipositor diameter, GSI, HSI, fecundity, oocytes weight, and ripeness [44, 45]. However, this finding showed that the proximate content was comparatively higher in the oocytes of both fish groups as compared to their liver. During gametogenesis, embryonic and early larval development of fish, protein, and lipids mobilize to the ovary from the liver [46]. Therefore, protein and lipid deposition in the oocytes was much higher than in the liver. These results have coincided with the findings of many previous literatures [4, 11, 47, 48]. Moreover, the ash content in the tissues of wild and tank-reared broodstock was not significantly varied. This could be due to almost identical minerals availability in the broodstock habitat environment. Kabir et al. [44] indicated that a higher level of inorganic phosphate is an essential ion for nucleic acid synthesis, ATP, and glycolysis in broodstock eggs. The amino acid composition in the liver and oocytes are vital molecules for the reproductive development of broodstock. In this observation, it was depicted that both wild and captive fish liver and oocytes had identical amino acids, except for lysine, phenylalanine, glycine, and glutamic acid. These amino acids were notably greater in the wild broodstock’s tissues as compared to the broodstock in captivity. The consumption of various natural foods and diverse environmental conditions might be the possible reasons for getting better results in wild fish. Further, the liver and oocytes may likely have a relationship since, during oocytes development, essential nutrients are transferred to the eggs from the liver through blood. Numerous researchers documented similar results in *Symphysodon aequifasciata* [49], *Gadus morhua* L [15]., *C. gariepinus* [35], *Oncorhynchus mykiss* [29]. Conversely, Ovissipour et al. [50] detected no remarkable variations in the amino acids of domestic and wild Beluga *Huso huso* ovary. The information on amino acid composition could be a valuable biomarker for identifying the specific amino acid in feed for the broodstock diet development of long-whiskered catfish aquaculture in captivity.

Ovarian histological analysis is one of the best ways to observe the sexual maturity and gonadal development of broodstock during the reproductive period. Numerous scientists [23, 51, 52] stated that histomorphological characteristics of the ovary provide important details on the stages of oocyte development and peak spawning season. The histological results showed that all the oocytes in the female ovary did not develop at a time, reflecting that this fish had an asynchronous ovarian oocyte development. Similar oocyte development patterns were also noted in *Trachurus trachurus* [53], *Pangasianodon hypophthalmus* [44], *Channa striatus* [54], *C. lazera* [55], and *C. gariepinus* [56, 57] and *S. aor* [3]. Moreover, the data on the frequency distribution of different stages of oocyte development revealed that captive fish ovaries had significantly higher and lower PNO and YVO, respectively, rather than wild fish ovaries. However, the percentage of YGO remained unchanged between the broodstock groups. Amzad et al. [22] and Sumon et al. [4] coincided with these results under different environmental conditions. Overall, the histological data revealed that wild fish might have an improved quality of eggs (especially GSI, oocyte weight, and ripeness) and also trigger the oocyte development stages, which in turn ripe the eggs quickly as compared to captive fish.

During the experiment, the highest value of temperature and rainfall was noted in April and August, respectively. The temperature and rainfall may have great impacts on the GSI, fecundity, and oocyte development of female broodstock [58, 59]. Furthermore, the reproduction ovarian oocytes development of fish may be affected by numerous factors, including rainfall, water temperature, and photoperiod, along with physicochemical features of water and features relating to the fish holding conditions [54, 60–64]. An overall variation of reproductive traits and ovarian development of broodstock in this study might be due to the environmental variables, habitat rich, and food.

## Conclusions

In summary, the intraspecific differences in some reproductive variables and two essential amino acids in liver and oocytes between wild and captive broodstock reveal variability in reproductive ovarian oocytes development, possibly due to a lack of environmental suitability, husbandry requirement, and nutritional status in food and diet. Additionally, the wild broodstock had more matured oocytes in their ovaries compared to the captive individuals, indicating that captivity may disrupt with the reproductive ovarian oocytes development progress of *S. aor*. Therefore, the present study highlights the need for an improvement in raising aquaculture technology for *S. aor*, which should include the formulation of a specific diet with amino acids supplementation, such as phenylalanine and lysine, to overcome the reproductive plasticity in captivity compared to wild breeders. Further study may be needed to determine the specific test diet for the development of *S. aor* broodstock in captive conditions.

## Data Availability

The data that support the findings of this study are available on request from the corresponding author [Muhammad Anamul Kabir and Zulhisyam Abdul Kari].
